# Development of a Publicly Available, Comprehensive Database of Fiber and Health Outcomes: Rationale and Methods

**DOI:** 10.1371/journal.pone.0156961

**Published:** 2016-06-27

**Authors:** Kara A. Livingston, Mei Chung, Caleigh M. Sawicki, Barbara J. Lyle, Ding Ding Wang, Susan B. Roberts, Nicola M. McKeown

**Affiliations:** 1 U.S. Department of Agriculture, Jean Mayer Human Nutrition Research Center on Aging, Tufts University, Boston, Massachusetts, United States of America; 2 Nutrition/Infection Unit, Department of Public Health and Community Medicine, Tufts University School of Medicine, Boston, Massachusetts, United States of America; 3 North American Branch of the International Life Sciences Institute, Washington, D.C., United States of America; 4 Friedman School of Nutrition Science and Policy, Tufts University, Boston, Massachusetts, United States of America; University of Chieti, ITALY

## Abstract

**Background:**

Dietary fiber is a broad category of compounds historically defined as partially or completely indigestible plant-based carbohydrates and lignin with, more recently, the additional criteria that fibers incorporated into foods as additives should demonstrate functional human health outcomes to receive a fiber classification. Thousands of research studies have been published examining fibers and health outcomes.

**Objectives:**

(1) Develop a database listing studies testing fiber and physiological health outcomes identified by experts at the Ninth Vahouny Conference; (2) Use evidence mapping methodology to summarize this body of literature. This paper summarizes the rationale, methodology, and resulting database. The database will help both scientists and policy-makers to evaluate evidence linking specific fibers with physiological health outcomes, and identify missing information.

**Methods:**

To build this database, we conducted a systematic literature search for human intervention studies published in English from 1946 to May 2015. Our search strategy included a broad definition of fiber search terms, as well as search terms for nine physiological health outcomes identified at the Ninth Vahouny Fiber Symposium. Abstracts were screened using a priori defined eligibility criteria and a low threshold for inclusion to minimize the likelihood of rejecting articles of interest. Publications then were reviewed in full text, applying additional a priori defined exclusion criteria. The database was built and published on the Systematic Review Data Repository (SRDR™), a web-based, publicly available application.

**Conclusions:**

A fiber database was created. This resource will reduce the unnecessary replication of effort in conducting systematic reviews by serving as both a central database archiving PICO (population, intervention, comparator, outcome) data on published studies and as a searchable tool through which this data can be extracted and updated.

## Introduction

Creating a database that captures all fiber types is challenging due to the large number of studies and diversity in fiber sources and composition [1]. The commonality to all fibers is the fact that they are non-digestible by endogenous enzymes; however, fiber is a group of structurally diverse compounds [2]. Fiber research is complex, in part because of the variety of terms used to describe fibers in publications, such as soluble fiber, fermentable fiber, viscous fiber, functional fiber, and added fibers, to name a few [2]. Notably, the term “fiber” includes both dietary fiber which is endogenous to food and functional fiber which is extracted or synthesized [3].

**Fig 1:**
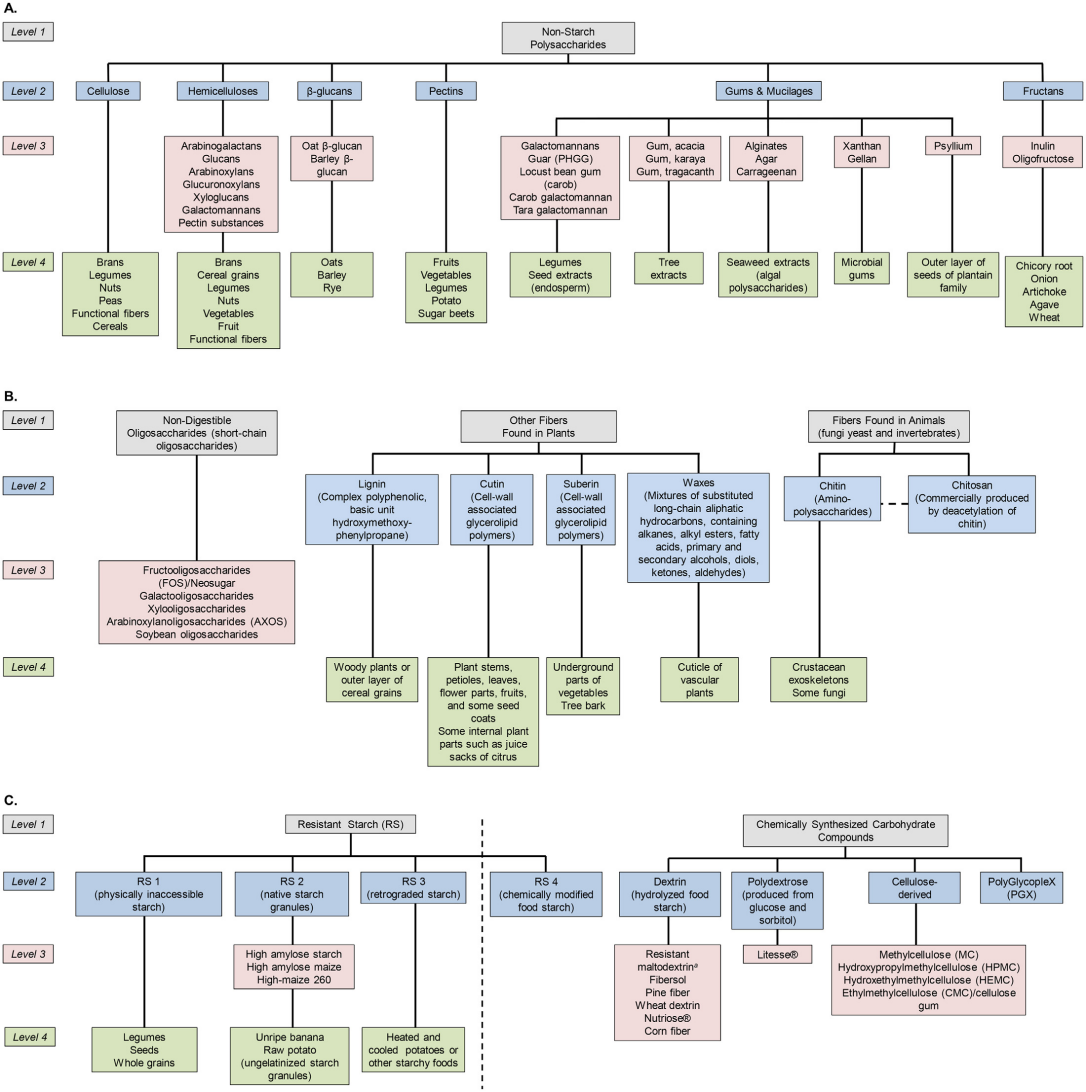
Schematic Illustration of Fiber Types and Levels of Classification. The figure is split into 3 panels (A-C) only to optimize space. Fibers are classified into groups (Level 1) and subgroups (Level 2) of physically related compounds. Isolated fibers, including some purified fibers in the form of commercially available supplements, are listed in Level 3 and food sources in Level 4. The dashed line in panel C is meant to indicate that RS4 could also be categorized with the other chemically synthesized fiber types. ^a^also referred to as maltodextrin (EEU, UK), indigestible dextrin (Asia, Japan), resistant dextrin (Brazil, UK), pyrodextrin.

As shown in the schematic illustration (Fig 1) reflective of the various levels of fiber, fiber can be a group of physically related compounds (e.g., non-starch polysaccharides (NSP) or oligosaccharides), synthetic or purified fibers in the form of supplements, individual isolated fiber (e.g., pectin and gum), enriched ingredients (e.g., oat bran, psyllium, or lupin kernel flour enriched breads), or described based on food sources (e.g. legumes or cereals). Additionally, fibers can be grouped based on their physical characteristics, including solubility (ability to dissolve or disperse in water), viscosity (ability to thicken or gel when mixed with fluids), and fermentability (ability to be fermented, or broken down, in the colon to produce short-chain fatty acids that can be used to yield energy) [2, 4]. Fibers can also be grouped by their physiological health outcomes. In addition to the above-mentioned complexities, fiber in the food matrix may function differently than the same fiber isolated and used as an additive, e.g. incorporated in a beverage, and fibers may undergo changes due to processing during cooking (e.g., heating), thereby changing the functional properties of the fiber.

The importance of fiber in human health has been increasingly recognized and, with research in this field moving at a fast pace, there is a demonstrated need for a database capturing and organizing published literature linking fiber to physiological health outcomes that can evolve over time with the growing body of research. The *Diet-Related Fibers and Human Health Outcomes Database* (Fiber Database) is a resource to assist health researchers and policy-makers in evaluating evidence linking fibers to specific physiological health outcomes in a quick and efficient manner. The database is maintained and updated by the authors, and database users may contact the corresponding author with inquiries. Annual updates to incorporate new literature into the database are funded and planned through 2017, at which time continuing updates will be discussed. In this paper, we describe the steps undertaken to create this database and the background methods for applications using the database.

### What is dietary fiber?

Fibers are found naturally in cereals, legumes, fruits, and vegetables, and the endogenous fiber in these foods has been linked to a number of physiological health outcomes. More recently, fiber is being added to food products. However, a universal definition of fiber has been historically difficult to establish, with an attempt to balance nutritional benefits and analytical methodology [5]. All of the various definitions are consistent in describing fibers as carbohydrate polymers that resist digestion in the small intestine and travel to the large intestine where they are at least partially fermented [6]. However, variation exists with regard to other components of the definitions of fibers accepted across the globe [6]. The two most widely used definitions, CODEX and Institute of Medicine (IOM), are described in further detail below.

At the 30th (Codex Alimentarius, 2008) and 31st (Codex Alimentarius, 2009) meetings of the Codex Committee on Nutrition and Foods for Special Dietary Uses, the definition of fiber and analytical methods for quantification of total fiber and individual specific components were agreed upon [1, 7, 8]. The CODEX Alimentarius definition describes dietary fiber as carbohydrate polymers of ten or more units that resist hydrolysis in the small intestine, although some national authorities have adopted somewhat different definitions allowing for inclusion of carbohydrates from 3 to 9 monomeric units in their definition. To this point, however, worldwide experts strongly agree on the inclusion of all indigestible carbohydrate oligomers and polymers with a degree of polymerization of 3 or higher [9, 10]. Dietary fiber under the CODEX definition falls into three categories: (1) fibers intrinsic to foods as consumed (natural food); (2) fibers that have been extracted from food materials by physical, enzymatic, or chemical means (food raw material); and (3) synthetic or modified fibers (synthetic). Fiber supplements are classifified under the latter two fiber categories (codex 2 or 3). Fibers classified as codex categories 2 or 3 must have accepted scientific evidence of some *physiological health benefit* in order to be considered fiber, as discussed in greater detail below.

The IOM’s definition of fiber is similar to the CODEX definition; however, the IOM definition distinguishes dietary fiber from functional fiber, with “fiber” being the sum of dietary and functional fibers [6]. “Dietary fiber” strictly refers to the “non-digestible carbohydrates and lignins that are intrinsic and intact in plants”‘ (i.e. intrinsic fiber), while all other”isolated, non-digestible carbohydrates that have beneficial physiological effects in humans” are referred to as “functional fibers” (i.e. added fiber) [11]. As such, fibers extracted from foods by physical, enzymatic, or chemical means or synthetic fibers fall into the functional fiber category and are not referred to as “dietary fiber” under the IOM definition. However, similar to the CODEX definition, these fibers must demonstrate a beneficial physiological health outcome to be classified as fiber. Fiber supplements are classified as “functional fibers” under the IOM definition. All isolated non-digestible carbohydrates will undergo reviews to ascertain status as a “fiber” for food labeling in the United States and Canada [12, 13].

### Defining a Physiological Health Outcome

At the Ninth Vahouny Fiber Symposium, experts in the field identified nine physiological health outcomes attributed to dietary fiber [9]. The health outcomes prioritized were: (1) total and LDL cholesterol, (2) post-prandial glucose and insulin, (3) blood pressure, (4) increased fecal bulk and laxation, (5) transit time, (6) colonic fermentation and short-chain fatty acid production, (7) modulation of colonic microflora, (8) weight loss, weight maintenance, and reduction in adiposity, and (9) increased satiety. These are the primary physiological health outcomes that could support fibers under categories 2 and 3 of the CODEX definition and IOM’s category of “functional” fiber, if demonstrated in epidemiological and randomized controlled (RCTs), with RCTs being considered the gold standard. Fibers that are intrinsic to foods in the form of naturally high-fiber foods (i.e., legumes, breakfast cereals, fruits and vegetables) have long been accepted to possess beneficial physiological health outcomes and should continue to be exposures of interest in future research. In addition, intervention studies isolating the singular effect of the fiber itself, rather than the food or diet, are needed to provide direct evidence linking specific fibers to health outcomes [11]. This need for differentiation between dietary fibers (endogenous to food) and functional fibers (extracted and/or synthesized from food) was highlighted in the IOM’s 2001 report [3].

While a large body of literature exists highlighting potential health benefits of fiber in relation to the nine physiological health outcomes identified by experts at the Vahouny Conference, the research varies widely in design, fiber dose, duration, and outcomes. Evidence reviews will need to focus on specific fibers and fiber applications to advance their categorization as “fiber.” Thus, there is a need to compile the literature in order to review and synthesize the existing evidence and examine fibers under the CODEX and IOM definitions. As such, the objectives of this database were to:

1. Systematically compile and provide access to primary, English-language, peer-reviewed science linking fiber intake in humans to one or more of nine physiological health outcomes;
2. Create a database to serve as a starting foundation of primary human literature for conducting evidence-based reviews and meta-analyses, focusing on intervention studies in humans;
3. Provide researchers with a tool to understand how different fibers are characterized in studies; and
4. Facilitate identification of opportunities and gaps in the current research.

The database is a tool that can be used to broadly summarize the body of research on fiber and health and used to facilitate evidence maps and systematic reviews. Evidence mapping involves capturing population, intervention, comparator, and outcome (PICO) information, thereby characterizing the existing research on a broad topic. In contrast to systematic reviews, evidence mapping does not include data or information on study findings or provide an in-depth, risk-of-bias evaluation of the included studies [14, 15]. It is considered a rapid, cost-effective methodology, that identifies *major research gaps* in the evidence base [14–20]. In fields outside of nutritional epidemiology, evidence mapping has been used to promote evidence-based decision making [15–17, 21, 22] and has particular utility when the research topic is complex and the amount of available data is large. One appreciable difference between evidence mapping and a systematic review is that evidence mapping provides a broader overview of existing research, and the goal is to produce a descriptive evidence map that then can be used for different purposes [23].

## Methods

### Building the Database

#### Step 1: Search Strategy

We aimed to identify existing literature published from 1946 to May 2015 examining the effects of fiber on at least one of the nine physiological health outcomes defined at the Vahouny Symposium [9]. To identify the relevant literature, we conducted a systematic, reproducible search in OVID Medline (OvidSP). See supplement (S1 Table) for the full search strategy. To capture literature of interest, our search strategy included a broad list of key terms related to the nine health outcomes combined with a search for dietary fiber and functional fiber, in addition to including both broad and specific fiber terms, and brand names of fiber supplements. We restricted our search to studies published in English. Cross-sectional studies, prevalence studies, case reports, bibliographies, and reviews were specifically excluded. The Medline search was restricted to identify studies conducted in humans or both humans and animals, with the exception of studies with microbiota outcomes (colonic fermentation and short-chain fatty acid production, modulation of colonic microflora) for which animal-only studies were also included. We used medical subject heading (MESH) terms and the multi-purpose search feature in OVID (.mp) to search all fields including title, abstract, subject heading words, and keyword heading words. MESH terms are assigned to manuscripts upon Medline indexing and are useful in allowing for retrieval of all references related to a particular topic, even if different terminology is used in the actual publication.

#### Step 2: Screening

We used the free, open source platform ABSTRAKR, developed by the Evidence-Based Practice Center at Brown University, to conduct an initial screening of all abstracts identified by the Ovid search [24]. During this phase, abstracts were identified as meeting inclusion criteria or not. Abstracts meeting criteria were defined as those meeting the a priori Medline search criteria and containing mention of both a fiber term and at least one of the nine physiological health outcomes in the abstract. During abstract screening additional decisions were made to exclude all animal-only, in vitro, or non-intervention studies, despite the fact that these studies were included in the initial search. Studies meeting these criteria (animal, in vitro, non-intervention/observational) were tagged, set aside, and not included in the fiber database.

The study team was instructed to use a low threshold for inclusion during screening in an attempt to include all potentially relevant studies and minimize discarding potential abstracts of interest. The first ten percent of abstracts were double-screened by different members of the study team. The double screening was conducted as a training process to ensure that screeners understood the inclusion and exclusion criteria. During training, screeners were instructed to review abstracts conservatively, to minimize the likelihood of rejecting abstracts of interest. During this training phase, 10% of those double-screened were identified as discordant, discussed in group training meetings, and reviewed by the principal investigator and research team for a final decision.

Full manuscripts were obtained for all abstracts classified as relevant during abstract screening, and a full text screen then was conducted. To better focus on results most relevant to the general population, we applied additional exclusion criteria during the full text screening phase. These additional exclusion criteria were as follows: (1) fiber was not orally ingested (i.e. administered intravenously, patients on enteral nutrition); (2) population was children <3 years of age; (3) population was pregnant and/or breastfeeding; (4) population had any type of cancer; (5) population had bowel disease (i.e., inflammatory bowel disease, Crohn’s disease, colostomy, etc); (6) population had renal failure; (7) population had other disease conditions (i.e., ileostomy patients); (8) intervention had no concurrent control group; (9) a defined fiber dose was not reported; (10) the intervention was not significantly controlled to measure the effect of the fiber; and (11) synbiotic studies. Reasons for exclusion during the full text screen phase were documented.

#### Step 3: Data Extraction and Publishing the Database

The database was built and published on the Systematic Review Data Repository (SRDR™), a web-based, publicly available application serving as both a central archive and data extraction tool. Version 1 of the database, containing literature published between 1946 and September 2013, was uploaded and made publicly available on April 27, 2015. Version 2 of the database, updated to contain literature through May 2015, was uploaded and made publicly available on December 31, 2015. Data were initially extracted, in most cases, by one extractor and included information on study design characteristics (i.e., design, blindness, study diet, level of feeding control); participant characteristics (i.e., age, gender, health status); fiber intervention details such as type of fiber administered, dose, and duration; placebo or comparator used with relevant dose and duration; and health outcomes examined, focusing on the nine Vahouny physiological health outcomes. Database users should verify all data from the original source to ensure that information extracted in the database is appropriately tailored to the needs of their individual summaries or evidence reviews.

The database captured a maximum of 4 fiber exposures, 4 comparators, and up to 8 physiological health outcomes per paper. Studies containing more than 4 fiber exposures were reviewed on a case-by-case basis, and similar exposures often were grouped for the purpose of database entry. The fiber exposure selected corresponded with the dose. Although the database was originally intended to capture only specific fiber types as exposures, upon beginning data extraction, we encountered papers where the intervention provided did not specify information on a specific fiber type. For example, the intervention was described generally only as a “high fiber diet,” or no information was provided on the specific fiber type in the intervention food, or the intervention contained a combination of fibers for which the dose provided was the total fiber. For such interventions, “dietary fiber” was entered as the fiber exposure. This is in contrast to “combination/mixture” exposures, where the specific components of the combination were specified, and a separate dose was provided for each of the fibers in the combination. If a study detailed more than 8 physiological health outcomes, data extractors were instructed to prioritize entering Vahouny outcomes. Since papers typically did not examine all nine Vahouny outcomes, the 8 outcome fields were sufficient for data capture. If additional room allowed after entry of all Vahouny outcomes, extractors could have included other study outcomes which were prioritized based on their emphasis in the paper. For example, outcomes that were highlighted in the abstract or focused on in the discussion were prioritized over those that were only briefly mentioned in the results.

In the event that data, such as age or fiber dose amount, was not explicitly stated in the paper, data extractors were instructed to calculate values when possible. Calculated values are indicated in the database by the use of a tilde (~) preceding the value. Finally, the database included a question on whether the exposure dose changed throughout the course of the study. In such cases, “yes” was selected, and the maximum dose was recorded in the dose field of the database. Ranges were entered into the database in cases where means were not available, such as for population age, BMI, or fiber dose. Some manuscripts contained multiple but distinct studies. In this case, manuscripts were entered into the database more than once. The publicly available database and detailed user manual can be found at the SRDR website’s published projects page (http://srdr.ahrq.gov/projects/published, “Diet-Related Fibers and Human Health Outcomes”; http://srdr.ahrq.gov/projects/716).

## Results

### Summary of Database Content

**Fig 2:**
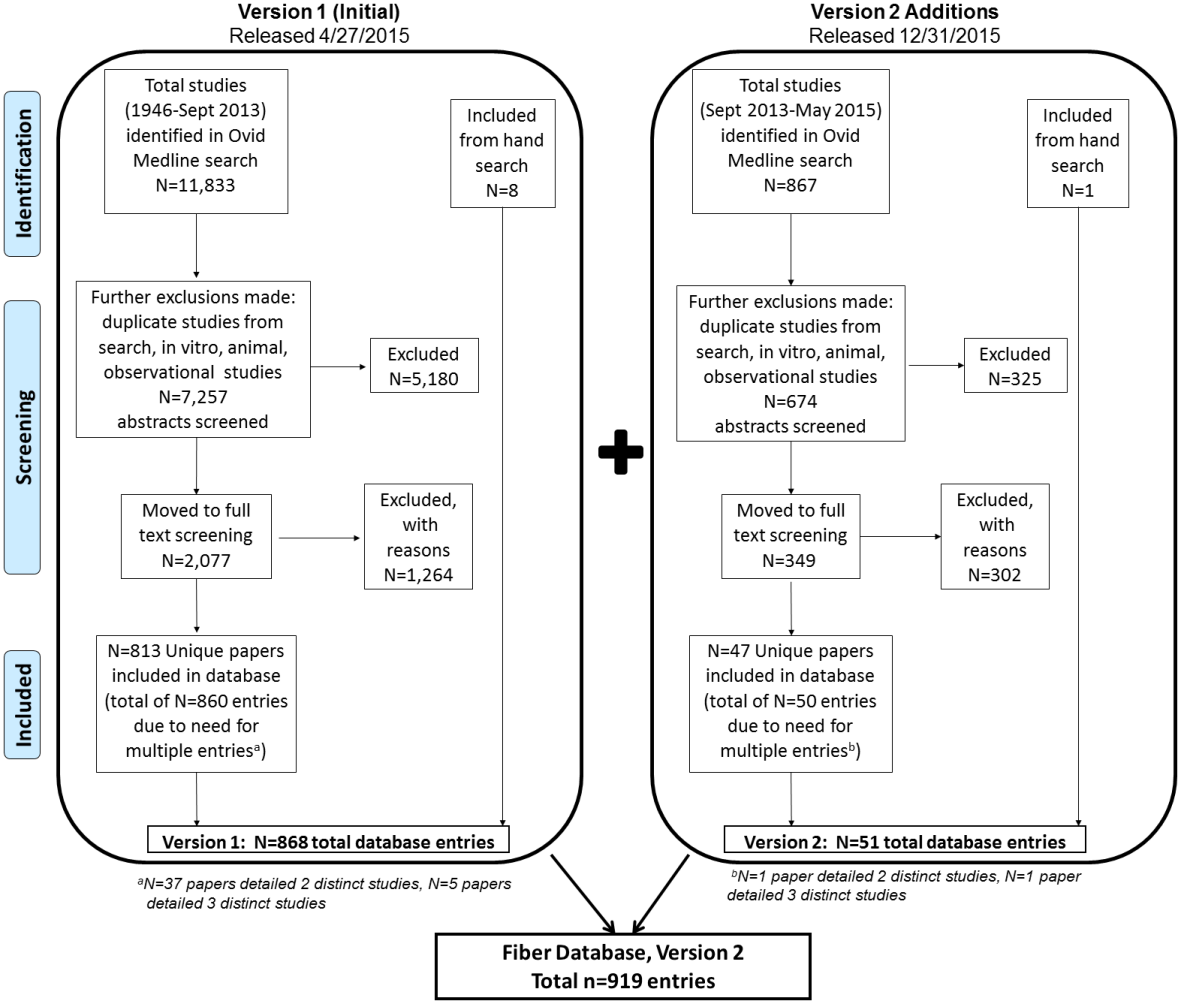
Flow Diagram of Studies.

We identified 7,931 abstracts (7,257 in version 1 + 674 in version 2) detailing human intervention studies, in English, containing a fiber exposure and Vahouny physiological health outcome (excluding in vitro, observational, and animal studies). Of the 7,931 abstracts screened, 2,426 (30.6%) were determined to broadly meet eligibility criteria. Of the 2,426, 860 were determined eligible for database inclusion after a detailed review of the full manuscript. Of the 860 included manuscripts, 38 detailed two distinct studies, and 6 detailed 3 distinct studies and, thus, were given multiple entries. In addition, 9 papers identified via hand search were included, yielding a final database with 919 total entries (Fig 2).

The 919 entries represent literature published from the year 1966 to 2015. As illustrated in Table 1, the majority of studies were randomized controlled (RCT) crossover (63.4%) or parallel (32.0%) designs, and only 34.7% of the studies clearly specified double-blinding. With respect to study populations, approximately 80% of studies were conducted in adults (20+ years), although studies among children less than 3 years of age were specifically excluded. Populations were predominately healthy (53.5%) or metabolically at risk (38.1%); however, crossover designs had a higher proportion of healthy subjects (64.1%) compared to parallel designs (30.6%). Over 40% of diets were isocaloric/maintenance diets and over 30% were acute feeding studies, where effects were measured after a single meal or day. Over 90% of studies were metabolic or partial metabolic studies. Approximately 36% of studies had a run-in period, 45% had a washout period, and 9.1% reported changing the dose of dietary fiber during the course of the study (data not shown).

**Table 1:** Characteristics of Studies Captured in Database (n = 919).

	Total	RCT Crossover	RCT Parallel	Other^1^
	919	583 (63.4%)	294 (32.0%)	42 (4.6%)
**Blinding**				
Unspecified	424 (46.1%)	307 (52.7%)	86 (29.2%)	31 (73.8%)
Double-blind	319 (34.7%)	158 (27.1%)	156 (53.1%)	5 (11.9%)
Single-blind	129 (14.0%)	94 (16.1%)	32 (10.9%)	3 (7.1%)
Not blinded	42 (4.6%)	22 (3.8%)	17 (5.8%)	3 (7.1%)
Other^2^	5 (0.5%)	2 (0.3%)	3 (1.0%)	0 (0.0%)
**Age Category**				
Adults (20+ y)	733 (79.8%)	461 (79.1%)	237 (80.6%)	35 (83.3%)
Adults & Adolescents (12–20+ y)	153 (16.6%)	102 (17.5%)	46 (15.6%)	5 (11.9%)
Other^3^	33 (3.6%)	20 (3.4%)	11 (3.7%)	2 (4.8%)
**Study Diet**				
Isocaloric/maintenance	416 (45.3%)	227 (38.9%)	170 (57.8%)	19 (45.2%)
Acute feeding study	288 (31.3%)	262 (44.9%)	11 (3.7%)	15 (35.7%)
Unspecified	143 (15.6%)	72 (12.3%)	66 (22.4%)	5 (11.9%)
Hypocaloric	51 (5.5%)	12 (2.1%)	37 (12.6%)	2 (4.8%)
Other^4^	21 (2.3%)	10 (1.7%)	10 (3.4%)	1 (2.4%)
**Level of Feeding Control**				
Food partially provided (partial metabolic)	503 (54.7%)	276 (47.3%)	213 (72.4%)	14 (33.3%)
All food provided (metabolic)	340 (37.0%)	287 (49.2%)	29 (9.9%)	24 (57.1%)
Food recommended	31 (3.4%)	6 (1.0%)	22 (7.5%)	3 (7.1%)
Dietary guidance with supplement or fiber treatment provided	28 (3.0%)	4 (0.7%)	24 (8.2%)	0 (0.0%)
Unspecified/other^5^	17 (1.9%)	10 (1.7%)	6 (2.0%)	1 (2.4%)
**Baseline Health**				
Healthy	492 (53.5%)	374 (64.1%)	90 (30.6%)	28 (66.7%)
Metabolically at risk	350 (38.1%)	166 (28.5%)	173 (58.8%)	11 (26.2%)
GI condition^6^	29 (3.2%)	11 (1.9%)	18 (6.1%)	0 (0.0%)
Mixed population	40 (4.3%)	29 (5.0%)	9 (3.1%)	2 (4.8%)
Other^7^	8 (0.9%)	3 (0.5%)	4 (1.4%)	1 (2.4%)

^1^ includes controlled trials with unspecified randomization, non-randomized trials, combined crossover and randomized controlled trial, and switch-back design

^2^ includes mixture of double/single blind, allocation-concealed, time-blinded, and triple blind

^3^ Includes combined populations of adults, adolescents, and/or children (3–11 y), as well as n = 3 studies where age information was not provided

^4^ includes AHA and NCEP diets, restricted diets (ie. carbohydrates, energy, fat), low-fiber background diets, and combinations of maintenance or weight loss diets based on individual participant BMI

^5^ includes mixture of food provided and food partially provided, mixture of food partially provided and food recommended, and not specified

^6^ includes constipation, digestive problems, diverticular disease, fecal incontinence, loose stools, and hemorrhoids

^7^ includes hospitalized for orthopedic surgery, in-patients prescribed antibiotics, recently removed colonic adenomas, or not reported

The 919 entries detailed a total of 1483 fiber exposures. Of these 1483, guar gum was the most frequently studied exposure (8.7%). Combinations/mixtures of more than one fiber (7.5%) and dietary fiber (6.5%) comprised a total of 14% of exposures. Of the 111 combinations/mixtures, 16 (14.4%) contained some form of guar. Exposures under the general classification of ‘dietary fiber’ were largely whole diet interventions where the goal was to increase total fiber through intake of high fiber foods, and doses provided were generally for total dietary fiber intake. Psyllium, which included psyllium hydrophilic mucilloid (Metamucil), psyllium seed husk, ispaghula husk, ispaghula, and isabgol, was also a frequently studied exposure (6.7%). Table 2 displays characteristics of studies on these top four reported fiber types. Characteristics are largely similar to those of the overall database (Table 1). Notably, though, a high proportion of studies on guar gum were acute (56.6%), whereas only 31.3% of studies in the overall database were acute. Resistant starches (4.9% of database exposures), including resistant starch types 1–4, resistant wheat starch, and high amylose starch, were frequently studied fiber types. Other top individual fibers studied included wheat bran (4.4%), oat β-glucan (3.9%), and oat bran (3.1%).

**Table 2:** Characteristics of Studies on Top Four Reported Fiber Types in Database (n = 437).

	Total	Guar Gum	Combination/Mixture	Psyllium	Dietary Fiber
	437	129 (8.7)	111 (7.5)	100 (6.7)	97 (6.5)
**Study Design**					
RCT Crossover	276 (63.2)	97 (75.2)	66 (59.5)	53 (53.0)	60 (61.9)
RCT Parallel	131 (30.0)	22 (17.1)	40 (36.0)	42 (42.0)	27 (27.8)
Other^1^	30 (6.9)	10 (7.7)	5 (4.5)	5 (5.0)	10 (10.3)
**Blinding**					
Unspecified	241 (55.1)	80 (62.0)	45 (40.5)	39 (39.0)	77 (79.4)
Double-blind	131 (30.0)	36 (27.9)	54 (48.7)	33 (33.0)	8 (8.2)
Single-blind	40 (9.1)	8 (6.2)	10 (9.0)	18 (18.0)	4 (4.1)
Not blinded	24 (5.5)	4 (3.1)	2 (1.8)	10 (10.0)	8 (8.2)
Other^2^	1 (0.2)	1 (0.8)	0 (0.0)	0 (0.0)	0 (0.0)
**Age Category**					
Adults (20+ y)	331 (75.7)	100 (77.5)	75 (67.6)	76 (76.0)	80 (82.5)
Adults & Adolescents (12–20+ y)	87 (19.9)	22 (17.1)	31 (27.9)	20 (20.0)	14 (14.4)
Other^3^	19 (4.3)	7 (5.4)	5 (4.5)	4 (4.0)	3 (3.1)
**Study Diet**					
Isocaloric/maintenance	171 (39.1)	35 (27.1)	38 (34.2)	53 (53.0)	45 (46.4)
Acute feeding study	170 (38.9)	73 (56.6)	48 (43.2)	17 (17.0)	32 (33.0)
Unspecified	65 (14.9)	15 (11.6)	13 (11.7)	26 (26.0)	11 (11.3)
Hypocaloric	23 (5.3)	5 (3.9)	9 (8.1)	2 (2.0)	7 (7.2)
Other^4^	8 (1.8)	1 (0.8)	3 (2.7)	2 (2.0)	2 (2.1)
**Level of Feeding Control**					
Food partially provided (partial metabolic)	183 (41.9)	57 (44.2)	47 (42.3)	61 (61.0)	18 (18.6)
All food provided (metabolic)	200 (45.8)	69 (53.5)	53 (47.8)	25 (25.0)	53 (54.6)
Food recommended	23 (5.3)	0 (0.0)	3 (2.7)	2 (2.0)	18 (18.6)
Dietary guidance with supplement or fiber treatment provided	18 (4.1)	1 (0.8)	5 (4.5)	9 (9.0)	3 (3.1)
Unspecified/other^5^	13 (3.0)	2 (1.6)	3 (2.7)	3 (3.0)	5 (5.1)
**Baseline Health**					
Healthy	199 (45.5)	60 (46.5)	60 (54.1)	39 (39.0)	40 (41.2)
Metabolically at risk	202 (46.2)	59 (45.7)	45 (40.5)	50 (50.0)	48 (49.5)
GI condition^6^	17 (3.9)	2 (1.6)	3 (2.7)	9 (9.0)	3 (3.1)
Mixed population	16 (3.7)	8 (6.2)	1 (0.9)	2 (2.0)	5 (5.1)
Other^7^	3 (0.7)	0 (0.0)	2 (1.8)	0 (0.0)	1 (1.0)

^1^ includes controlled trials with unspecified randomization, non-randomized trials, combined crossover and randomized controlled trial, and switch-back design

^2^ time-blinded

^3^ includes adolescents and studies where age information was not provided

^4^ includes AHA diet, weight loss diets, and combinations of maintenance or weight loss diets based on individual participant BMI

^5^ includes mixture of food provided and food partially provided, mixture of food partially provided and food recommended, and not specified

^6^ includes constipation, diverticular disease, fecal incontinence, loose stools, and hemorrhoids

^7^ includes recently removed colonic adenomas, or not reported

The 919 entries detailed 3,581 outcomes (Table 3). Total and LDL cholesterol (16.9%), HDL cholesterol or triglycerides (15.9%), although not specifically a Vahouny health outcome, and postprandial glycemia/insulinemia (14.5%) were the top three outcome groups studied.

**Table 3:** Frequency of Outcomes Captured by Database (n = 3,581).

Health Outcome	N (%)
Total & LDL cholesterol	606 (16.9%)
Lipids^1^	570 (15.9%)
Postprandial glycemia/insulinemia	520 (14.5%)
Glucose & insulin metabolism	348 (9.7%)
Weight/adiposity	330 (9.2%)
Satiety	315 (8.8%)
Fecal bulk/laxation	254 (7.1%)
Colonic fermentation/SCFA production	173 (4.8%)
Blood pressure	142 (4.0%)
Modulation of colonic microflora	134 (3.7%)
Transit time	116 (3.2%)
GI symptoms^2^	59 (1.6%)
Other, non-Vahouny outcomes^3^	14 (0.4)

^1^ HDL cholesterol and triglycerides (non-Vahouny specified outcomes)

^2^ including constipation, GI tolerance, abdominal pain, diarrhea, digestive symptoms, and general GI side effects and symptoms

^3^ i.e. bile acid kinetics, cholesterol absorption & synthesis, coagulation factor, micronutrient levels, micronutrient balance

## Discussion

The fiber database is built and published on the Systematic Review Data Repository (SRDR™), a web-based, publicly available application that is an easy-to-use tool for the extraction and management of data for systematic reviews or meta-analysis. The database contains PICO data to help users formulate and narrow the focus of their research question. It can serve as a quick and useful resource to link dietary fibers to physiological health outcomes and ensures the data extracted is derived from intervention trials. The US Food and Drug Administration has proposed a revision to the Nutrition and Supplement facts labels, and although it is noted there is “no specific chemical definition for dietary fiber,” they proposed adoption of the IOM’s definition of fiber and tentatively concluded “that a regulatory definition for dietary fiber should be one that emphasizes its physiological effect that is beneficial to human health” [25]. In this respect, this database may serve as a useful tool in reviewing the evidence on the physiological health benefits of fibers.

For research scientists, this database offers a great deal of flexibility. By customizing their own search criteria, they can use this database to reduce the number of papers to those specific to their research question. As such, the advantage of this database is that it saves time, yet ensures a high level of quality is maintained. The users of this database may contact the research group at Tufts if they need assistance in customizing their search. Annual updates are planned to be released by January of each year, thereby providing users with access to updated references on specific fibers.

This fiber database is a starting point for conducting systemic reviews on fiber and nine physiological health outcomes. It serves to save on sustantantial resources (time and money) in the early resource-intensive stages of conducting literature searches, screening, and extraction of PICO information. It will reduce the unnecessary replication of effort associated with a systematic review by serving as both a central database archiving summary data on published studies and a searchable tool through which this summary data can be extracted and updated annually. Users can employ this database for a preliminary scope of how much evidence is available on a specific fiber and outcome and, thereby, identify specific fiber-health outcome pairings with substantial evidence and determine gaps in the existing literature.

This database is the first to systematically and comprehensively capture published literature linking fiber to health outcomes. Users should be familiar with the limitations of this database. First, our electronic search strategy was restricted to the Medline database as a means of maintaining some level of quality control while capturing the majority of human research. Medline houses over 20 million references dating back to 1946, and indexed journals are reviewed and recommended by an NIH-chartered advisory committee. In addition, Medline is advantageous for conducting searches because it utilizes medical subject headings (MESH), controlled by the National Library of Medicine, to index citations and facilitate searching. If a systematic review is the objective, then secondary sources are recommended, such as Cochrane, CAB abstracts, EMBASE, and others as appropriate to the application. Users are encouraged to follow good evidence-based review practices, including secondary literature searches, and would be expected to review and revise inclusion/exclusion criteria, as well. The PRISMA statement may be used as a starting point for conducting systematic reviews and meta-analyses [26]. It should also be noted that our fiber database does not specifically assess study quality, and while the majority of the studies capture the isolated effects of fibers, some studies may be confounded by other attributes of the foods (such as low glycemic index).

With regard to missing literature, our search strategy also may have missed manuscripts if fiber and/or outcome terms were not included in the search fields (i.e., title, keywords, abstract). In an effort to reduce this likelihood, we developed an extensive list of search terms in discussion with the research team and experts in the field. Our search strategy included outcomes, as we specifically aimed to capture literature examining one of our nine physiological health outcomes. This differs from the traditional search strategy used in a systematic review in which search terms of outcomes are typically not included in an attempt to capture studies with “buried” outcomes.

The database captures information as it was reported in the published manuscript, and attempts were not made to contact authors. Thus, data extractors did not make assumptions about methodology, extracting only information that was clearly stated. If authors did not include information on methods (ie. specifying whether study was randomized or blinded), it will be listed as not reported or missing in the database. Since this database is intended to be used as a tool to identify relevant literature, fibers and physiological health outcomes studied are reported, but specific results are not. In addition, due to restrictions around time and resources, abstracts were largely single screened, and data extraction for the majority of entries was single (rather than duplicate) entry.

Finally, the database, currently housed in SRDR, is downloadable in the form of a Microsoft Excel spreadsheet, allowing users to modify or add fields (i.e. results) to their own downloaded version of the database to suit individual needs. With respect to more complex searches and evidence mapping, however, importing it into a statistical analysis package such as SAS, SPSS, or STATA is advised.

## Conclusion

Dennis Gordon, a recognized expert in the field of fiber, captured the complexity of “what is dietary fiber?” as follows: “Dietary Fiber is many things to many people. It is a concept, a hypothesis, a marketer’s bonanza, a unique complex of non-digestible carbohydrates, but most importantly an integral necessity of a normal functioning and healthy intestine”[27]. This sentiment necessitates the need for a tool that systematically captures and organizes the large scope of complex literature on fiber and health. Practically speaking, fiber definitions for many food components and ingredients rely on evidence linking specific fibers with one or more recognized physiological health outcomes. This database can be used as a starting point for needed evidence reviews and is scheduled for annual updates for at least three years to incorporate new literature. For researchers conducting systematic reviews and meta-analysis, this database includes information on controlled, metabolic studies designed to provide evidence linking dietary fibers to health outcomes. Specific applications for evidence reviews include food manufacturers developing fibers and fiber-enriched foods, agencies defining fiber for food labeling, and health researchers and organizations evaluating the health benefits of different fiber sources.

## Supporting Information

S1 Checklist Preferred Reporting Items for Systematic Reviews and Meta-Analyses (PRISMA). (DOCX)

S1 Table OVID Medline Search Stategy for Fiber Database. (PDF)
