# Supplementary material for: Development of a Publicly Available, Comprehensive Database of Fiber and Health Outcomes: Rationale and Methods
Source: PLoS One. 2016 Jun 27;11(6):e0156961. doi: 10.1371/journal.pone.0156961 (PMC4922652; doi:10.1371/journal.pone.0156961)
Supplement: S1 Table — (PDF) [file pone.0156961.s002.pdf]

S1 Table: Ovid Medline Search Strategy for Fiber Database

----

1 Dietary fiber.mp. or exp Dietary Fiber/

2 ("dietary fibre" or "functional fibre" or "added fibre").mp.

3 ("dietary fiber" or "functional fiber" or "added fiber").mp.

4 or/1-3

5 (Alginates or Alphacyclodextrin or Alpha-cyclodextrin or Arabinoxylan or Arabinoxylan-oligosaccharides or AXOS).mp.

6 aleurone.mp. [mp=title, abstract, original title, name of substance word, subject heading word, keyword heading word, protocol supplementary concept word, rare disease supplementary concept word, unique identifier]

7 ("Barley Bran" or "Barley grain" or Beta-glucans).mp.

8 (Carrageenans or Cellulose or Chitin or Chitosan or "Corn bran").mp.

9 resistant maltodextrin.mp.

10 (Fructan$ or Fructooligosaccharide$ or Fructo-oligosaccharide$ or "short chain fructo-oligosaccharide" or "short chain fructo oligosaccharide").mp.

11 (Galactooligosaccharide$ or Gum$ or Galactomannan or Arabinogalactan).mp.

12 (Hemicelluloses or "Hydroxypropyl methylcellulose" or Inulin or "wheat dextrin" or dextrin or "resistant wheat starch" or "wheat starch").mp.

13 Inulin/

14 ("resistant starch" or sterculia or "Konjac mannan").mp.

15 (Legume or "bean fiber" or Lignin or "Locust bean" or Methylcellulose).mp.

16 ("Oat Bran" or Oligofructose or "Okra adj2 gum" or "guar adj2 gum").mp.

17 ("Pea fiber" or "pea hull" or Pectin or Polydextrose or "Potato fiber" or Psyllium or Ispaghula).mp.

18 "Resistant dextrin$".mp.

19 Resistant Starch.mp.

20 "Chemically modified starch".mp.

21 ("gum adj2 arabic" or "high amylose starch" or "karaya adj2 gum" or "carob adj2 gum").mp. [mp=title, abstract, original title, name of substance word, subject heading word, keyword heading word, protocol supplementary concept word, rare disease supplementary concept word, unique identifier]

22 ("Rice Bran" or "Rye fibre" or "Rye fiber" or "Rye Bran" or "Soluble corn fiber" or "Soy fiber" or "Sugar Beet" or sugarbeet or "Sugar cane fiber" or "Tara gum" or "Wheat Bran" or "Xanthan gum" or Xylans or Xyloglucans).mp.

23 ("retrograded resistant starch" or "resistant starch type$" or "high amylase starch").mp. [mp=title, abstract, original title, name of substance word, subject heading word, keyword heading word, protocol supplementary concept word, rare disease supplementary concept word, unique identifier]

24 ("sugar beet fiber" or "sugar beet fibre" or "sugarbeet fiber" or "sugarbeet fibre").mp. [mp=title, abstract, original title, name of substance word, subject heading word, keyword heading word, protocol supplementary concept word, rare disease supplementary concept word, unique identifier]

25 or/5-24

26 (Benefiber or Citrucel or FiberChoice or Fibersure or Hi-Maize or Konsyl or Fibersol or Metamucil or Normacol).tw.

27 (Actilight or Meiologio or NutraFlora or neosugar or Normafib or Orafti or Synergy1).tw.

28 (Litesse or Nutriose or Novelose or Fibersym).tw.

29 (actistar or BranaVita or Fibrulose or GrainWise or Oliggo-Fiber or Oliggofiber or Fibruline or "metamucil clear and natural" or "metamucil clear & natural" or naturaflora or normafibe or "orafti inulin" or "beneo synergy 1" or "beneo synergy" or "novelose 330" or novelose).tw.

30 or/26-29

31 4 or 25 or 30

32 randomized controlled trial.pt.

33 controlled clinical trial.pt.

34 randomized controlled trials/

35 Random Allocation/

36 Double-blind Method/

37 Single-Blind Method/

38 clinical trial.pt.

39 Clinical Trials.mp. or exp Clinical Trials/

40 (clinic$ adj25 trial$).tw.

41 ((singl$ or doubl$ or trebl$ or tripl$) adj (mask$ or blind$)).tw.

42 Placebos/

43 placebo$.tw.

44 random$.tw.

45 trial$.tw.

46 (randomized control trial or clinical control trial).sd.

47 (latin adj square).tw.

48 Comparative Study.tw. or Comparative Study.pt.

49 exp Evaluation studies/

50 Follow-Up Studies/

51 Prospective Studies/

52 (control$ or prospectiv$ or volunteer$).tw.

53 Cross-Over Studies/

54 exp Case-Control Studies/ or Control Groups/ or Matched-Pair Analysis/ or ((case* adj5 control*) or (case adj3 comparison*) or control group*).ti,ab.

55 cohort.ti,ab. or exp Cohort Studies/ or longitudinal.ti,ab. or prospective.ti,ab.

56 exp cohort studies/ or exp prospective studies/ or exp epidemiologic studies/ or exp case-control studies/

57 (cohort or prospective or longitudinal or follow-up or followup or registry).af.

58 case-control.af. or (case adj10 control).tw.

59 ep.fs.

60 exp intervention studies/

61 dietary intervention.mp.

62 exp Cross-Sectional Studies/ or cross-sectional.ti,ab. or "prevalence study".ti,ab.

63 (or/32-61) not 62

64 31 and 63

65 limit 64 to (addresses or bibliography or biography or case reports or comment or congresses or dictionary or directory or duplicate publication or editorial or guideline or interview or lectures or legal cases or legislation or letter or news or newspaper article or "review")

66 64 not 65

67 limit 66 to english language

68 limit 67 to animals

69 67 not 68

70 exp Cholesterol, HDL/ or exp Cholesterol/ or exp Cholesterol, LDL/ or exp Cholesterol Esters/

71 exp Hypercholesterolemia/

72 Hypercholester*mia.mp.

73 exp Hyperlipidemias/ or exp Dyslipidemias/

74 (Dyslipidemia$ or Dyslipoproteinemia$).mp.

75 (Hyperlipemia$ or Lipidemia$ or Lipemia$).mp.

76 exp Receptors, LDL/

77 (Oxidized adj2 LDL).mp. [mp=title, abstract, original title, name of substance word, subject heading word, keyword heading word, protocol supplementary concept word, rare disease supplementary concept word, unique identifier]

78 (Acetyl-LDL or Acetyl LDL).mp.

79 Scavenger Receptor.mp. or exp Receptors, Scavenger/

80 exp Lipoproteins, LDL/ or exp Lipoproteins/

81 ("Low Density Lipoprotein Cholesterol" or "beta-Lipoprotein Cholesterol" or "Cholesterol, beta-Lipoprotein" or "beta Lipoprotein Cholesterol" or "LDL Cholesterol" or "Cholesteryl Linoleate, LDL" or "LDL Cholesteryl Linoleate").mp.

82 ("low-density adj2 lipoprotein$" or "beta-lipoprotein$" or "beta adj2 lipoprotein$" or LDL-1 or LDL-2 or LDL1 or LDL2).mp.

83 ("LDL adj2 receptor$" or "(LDL adj2 receptor$) adj2 lipoprotein$" or "(low adj2 density) adj2 (lipoprotein adj2 receptor$)" or "low density lipoprotein receptor$" or "LDL receptor$").mp. [mp=title, abstract, original title, name of substance word, subject heading word, keyword heading word, protocol supplementary concept word, rare disease supplementary concept word, unique identifier]

84 ("oxidized LDL receptor" or "oxidized adj2 (LDL adj2 receptor$)" or "oxidized adj2 LDL" or "Ox adj2 (LDL adj2 receptor$)" or "ox-LDL").mp. [mp=title, abstract, original title, name of substance word, subject heading word, keyword heading word, protocol supplementary concept word, rare disease supplementary concept word, unique identifier]

85 ("receptor$ adj2 scavenger" or "(acetyl adj2 LDL) adj2 receptor$" or "(acetyl-LDL) adj2 receptor$" or "scavenger adj2 receptor" or "acetylated adj2 receptor" or "acetylated LDL receptor" or "(LDL receptor) adj2 acetylated" or "macrophage adj receptor$" or "macrophage scavenger receptor$" or "receptor adj2 (macrophage scavenger)").mp. [mp=title, abstract, original title, name of substance word, subject heading word, keyword heading word, protocol supplementary concept word, rare disease supplementary concept word, unique identifier]

86 (circulating adj2 lipoprotein$).mp. [mp=title, abstract, original title, name of substance word, subject heading word, keyword heading word, protocol supplementary concept word, rare disease supplementary concept word, unique identifier]

87 or/70-86

88 exp Constipation/

89 dyschezia.mp.

90 (colonic adj2 inertia).mp. [mp=title, abstract, original title, name of substance word, subject heading word, keyword heading word, protocol supplementary concept word, rare disease supplementary concept word, unique identifier]

91 exp Defecation/

92 (stool adj2 retention).mp. [mp=title, abstract, original title, name of substance word, subject heading word, keyword heading word, protocol supplementary concept word, rare disease supplementary concept word, unique identifier]

93 (bowel adj2 movement$).mp. [mp=title, abstract, original title, name of substance word, subject heading word, keyword heading word, protocol supplementary concept word, rare disease supplementary concept word, unique identifier]

94 (fecal adj3 weight$).mp. [mp=title, abstract, original title, name of substance word, subject heading word, keyword heading word, protocol supplementary concept word, rare disease supplementary concept word, unique identifier]

95 laxation.mp.

96 (stool adj2 consisten$).mp. [mp=title, abstract, original title, name of substance word, subject heading word, keyword heading word, protocol supplementary concept word, rare disease supplementary concept word, unique identifier]

97 (stool adj2 frequenc$).mp. [mp=title, abstract, original title, name of substance word, subject heading word, keyword heading word, protocol supplementary concept word, rare disease supplementary concept word, unique identifier]

98 or/88-97

99 (transit adj2 time).mp. [mp=title, abstract, original title, name of substance word, subject heading word, keyword heading word, protocol supplementary concept word, rare disease supplementary concept word, unique identifier]

100 ((colonic or colon or intestinal or gastrointestinal or bowel or gut) adj2 "transit time").mp. [mp=title, abstract, original title, name of substance word, subject heading word, keyword heading word, protocol supplementary concept word, rare disease supplementary concept word, unique identifier]

101 (stool adj frequenc$).mp. [mp=title, abstract, original title, name of substance word, subject heading word, keyword heading word, protocol supplementary concept word, rare disease supplementary concept word, unique identifier]

102 or/99-101

103 (colonic adj fermentation$).mp. [mp=title, abstract, original title, name of substance word, subject heading word, keyword heading word, protocol supplementary concept word, rare disease supplementary concept word, unique identifier]

104 fermentations.mp. or exp Fermentation/

105 exp Fatty Acids, Volatile/

106 fatty acids, short chain.mp.

107 (acetic adj2 acid$).mp. [mp=title, abstract, original title, name of substance word, subject heading word, keyword heading word, protocol supplementary concept word, rare disease supplementary concept word, unique identifier]

108 ("fatty acid$" adj2 "short-chain").mp. [mp=title, abstract, original title, name of substance word, subject heading word, keyword heading word, protocol supplementary concept word, rare disease supplementary concept word, unique identifier]

109 ("fatty acid$" adj2 "short chain").mp. [mp=title, abstract, original title, name of substance word, subject heading word, keyword heading word, protocol supplementary concept word, rare disease supplementary concept word, unique identifier]

110 (ester$ adj2 "acetic acid$").mp. [mp=title, abstract, original title, name of substance word, subject heading word, keyword heading word, protocol supplementary concept word, rare disease supplementary concept word, unique identifier]

111 exp Butyrates/

112 (n adj butyrate$).mp. [mp=title, abstract, original title, name of substance word, subject heading word, keyword heading word, protocol supplementary concept word, rare disease supplementary concept word, unique identifier]

113 (butyric adj2 acid$).mp. [mp=title, abstract, original title, name of substance word, subject heading word, keyword heading word, protocol supplementary concept word, rare disease supplementary concept word, unique identifier]

114 (butanoic adj2 acid$).mp. [mp=title, abstract, original title, name of substance word, subject heading word, keyword heading word, protocol supplementary concept word, rare disease supplementary concept word, unique identifier]

115 exp Caproates/

116 (hexanoate$ or "hexanoic adj2 acid$" or "caproic adj2 acid$" or "capronic adj2 acid$").mp. [mp=title, abstract, original title, name of substance word, subject heading word, keyword heading word, protocol supplementary concept word, rare disease supplementary concept word, unique identifier]

117 exp Propionates/

118 (propanoate$ or "propionic adj2 acid$" or "propanoic adj2 acid$").mp. [mp=title, abstract, original title, name of substance word, subject heading word, keyword heading word, protocol supplementary concept word, rare disease supplementary concept word, unique identifier]

119 exp Valerates/

120 pentanoate$.mp.

121 bifidobacteria.mp. or exp Bifidobacterium/

122 exp Acetates/

123 or/103-122

124 (modulation adj2 "colonic microflora").mp. [mp=title, abstract, original title, name of substance word, subject heading word, keyword heading word, protocol supplementary concept word, rare disease supplementary concept word, unique identifier]

125 exp Metagenome/

126 microbiome$.mp.

127 exp Lactobacillus helveticus/ or lactobacilli.mp. or exp Lactobacillus casei/ or exp Lactobacillus leichmannii/ or exp Lactobacillus reuteri/ or exp Lactobacillus brevis/ or exp Lactobacillus plantarum/ or exp Lactobacillus delbrueckii/ or exp Lactobacillus acidophilus/ or exp Lactobacillus/ or exp Lactobacillus rhamnosus/ or exp Lactobacillus fermentum/

128 (microbiota$ or "human$ adj2 microbiome$").mp. [mp=title, abstract, original title, name of substance word, subject heading word, keyword heading word, protocol supplementary concept word, rare disease supplementary concept word, unique identifier]

129 ((colon$ or intestinal or gastrointestinal or bowel or gut) adj2 (microflora$ or bacteria$ or microorganism$)).mp. [mp=title, abstract, original title, name of substance word, subject heading word, keyword heading word, protocol supplementary concept word, rare disease supplementary concept word, unique identifier]

130 (microbial adj2 (population$ or communit$ or composition$)).mp. [mp=title, abstract, original title, name of substance word, subject heading word, keyword heading word, protocol supplementary concept word, rare disease supplementary concept word, unique identifier]

131 (flora$ adj2 (autochthonous or indigenous or allochthonous or transient)).mp. [mp=title, abstract, original title, name of substance word, subject heading word, keyword heading word, protocol supplementary concept word, rare disease supplementary concept word, unique identifier]

132 (pH adj2 (fecal or stool)).mp. [mp=title, abstract, original title, name of substance word, subject heading word, keyword heading word, protocol supplementary concept word, rare disease supplementary concept word, unique identifier]

133 (or/124-132) or 121

134 (weight adj2 (loss or maintenance or reduction)).mp. [mp=title, abstract, original title, name of substance word, subject heading word, keyword heading word, protocol supplementary concept word, rare disease supplementary concept word, unique identifier]

135 exp Weight Loss/

136 exp Body Weight Changes/

137 exp Body Weight/

138 (reduction$ adj2 adiposity).mp. [mp=title, abstract, original title, name of substance word, subject heading word, keyword heading word, protocol supplementary concept word, rare disease supplementary concept word, unique identifier]

139 exp Weight Gain/

140 exp Obesity/ or exp Overweight/

141 (obesit$ adj2 morbid).mp. [mp=title, abstract, original title, name of substance word, subject heading word, keyword heading word, protocol supplementary concept word, rare disease supplementary concept word, unique identifier]

142 exp Obesity Hypoventilation Syndrome/

143 (pickwickian adj2 syndrome$).mp. [mp=title, abstract, original title, name of substance word, subject heading word, keyword heading word, protocol supplementary concept word, rare disease supplementary concept word, unique identifier]

144 exp Obesity, Abdominal/

145 ((central adj2 obesit$) or (visceral adj2 obesit$)).mp. [mp=title, abstract, original title, name of substance word, subject heading word, keyword heading word, protocol supplementary concept word, rare disease supplementary concept word, unique identifier]

146 exp Body Constitution/

147 (body weights and measures).mp. [mp=title, abstract, original title, name of substance word, subject heading word, keyword heading word, protocol supplementary concept word, rare disease supplementary concept word, unique identifier]

148 (body adj2 (weight$ or measure$)).mp. [mp=title, abstract, original title, name of substance word, subject heading word, keyword heading word, protocol supplementary concept word, rare disease supplementary concept word, unique identifier]

149 exp Body Fat Distribution/

150 ("body fat" adj2 patterning).mp. [mp=title, abstract, original title, name of substance word, subject heading word, keyword heading word, protocol supplementary concept word, rare disease supplementary concept word, unique identifier]

151 (fat adj2 patterning).mp. [mp=title, abstract, original title, name of substance word, subject heading word, keyword heading word, protocol supplementary concept word, rare disease supplementary concept word, unique identifier]

152 exp Adiposity/

153 exp Body Mass Index/

154 (quetelet$ adj2 index).mp. [mp=title, abstract, original title, name of substance word, subject heading word, keyword heading word, protocol supplementary concept word, rare disease supplementary concept word, unique identifier]

155 exp Body Size/

156 exp Waist Circumference/

157 exp Waist-Hip Ratio/

158 exp Skinfold Thickness/

159 or/134-158

160 exp Satiation/

161 exp Satiety Response/

162 (appetite adj2 alteration$).mp. [mp=title, abstract, original title, name of substance word, subject heading word, keyword heading word, protocol supplementary concept word, rare disease supplementary concept word, unique identifier]

163 exp Appetite Regulation/

164 ("intake regulation$" adj2 food$).mp. [mp=title, abstract, original title, name of substance word, subject heading word, keyword heading word, protocol supplementary concept word, rare disease supplementary concept word, unique identifier]

165 ("food intake$" adj2 regulation$).mp. [mp=title, abstract, original title, name of substance word, subject heading word, keyword heading word, protocol supplementary concept word, rare disease supplementary concept word, unique identifier]

166 exp Energy Intake/

167 (caloric adj2 intake$).mp. [mp=title, abstract, original title, name of substance word, subject heading word, keyword heading word, protocol supplementary concept word, rare disease supplementary concept word, unique identifier]

168 (satiety-related adj2 hormone$).mp. [mp=title, abstract, original title, name of substance word, subject heading word, keyword heading word, protocol supplementary concept word, rare disease supplementary concept word, unique identifier]

169 ((satiety adj2 hormone$) or (hunger adj2 hormone$)).mp. [mp=title, abstract, original title, name of substance word, subject heading word, keyword heading word, protocol supplementary concept word, rare disease supplementary concept word, unique identifier]

170 exp Ghrelin/

171 exp Leptin/

172 ("total peptide tyrosine" or "total peptide tyrosine-tyrosine" or "tyrosine" or "PYY").mp. [mp=title, abstract, original title, name of substance word, subject heading word, keyword heading

word, protocol supplementary concept word, rare disease supplementary concept word, unique identifier]

173 ("total glucagon-like peptide-1" or GLP-1 or GLP1 or "glucagon like peptide 1").mp. [mp=title, abstract, original title, name of substance word, subject heading word, keyword heading word, protocol supplementary concept word, rare disease supplementary concept word, unique identifier]

174 or/160-173

175 blood glucose.mp. or exp Blood Glucose/

176 (blood adj2 sugar).mp. [mp=title, abstract, original title, name of substance word, subject heading word, keyword heading word, protocol supplementary concept word, rare disease supplementary concept word, unique identifier]

177 exp Hemoglobin A, Glycosylated/

178 (hemoglobin$ adj2 glycated).mp. [mp=title, abstract, original title, name of substance word, subject heading word, keyword heading word, protocol supplementary concept word, rare disease supplementary concept word, unique identifier]

179 hyperglycemia$.mp. or exp Hyperglycemia/

180 glucose intolerance$.mp. or exp Glucose Intolerance/

181 (glucose adj2 intolerance$).mp. [mp=title, abstract, original title, name of substance word, subject heading word, keyword heading word, protocol supplementary concept word, rare disease supplementary concept word, unique identifier]

182 "glucose tolerance test$".mp. or exp Glucose Tolerance Test/

183 ("oral glucose tolerance$" or ogtt or "intravenous glucose tolerance$").mp. [mp=title, abstract, original title, name of substance word, subject heading word, keyword heading word, protocol supplementary concept word, rare disease supplementary concept word, unique identifier]

184 insulin.mp. or exp Insulin/

185 exp Hyperinsulinism/ or hyperinsulin*.mp.

186 exp Insulin Resistance/

187 (insulin adj resistance).mp. [mp=title, abstract, original title, name of substance word, subject heading word, keyword heading word, protocol supplementary concept word, rare disease supplementary concept word, unique identifier]

188 (insulin adj sensitivity).mp. [mp=title, abstract, original title, name of substance word, subject heading word, keyword heading word, protocol supplementary concept word, rare disease supplementary concept word, unique identifier]

189 proinsulin.mp. or exp Proinsulin/

190 "c peptide".mp. or exp C-Peptide/

191 connecting peptide/ or proinsulin c-peptide/ or proinsulin c peptide/ or c-peptide, proinsulin/ or c peptide proinsulin.mp. [mp=title, abstract, original title, name of substance word, subject heading word, keyword heading word, protocol supplementary concept word, rare disease supplementary concept word, unique identifier]

192 receptor, insulin.mp. or exp Receptor, Insulin/

193 glycemic ind*.mp.

194 exp Glycemic Index/

195 exp Diabetes Mellitus, Type 2/

196 or/175-194

197 or/195-196

198 exp Hypertension/

199 blood pressure.mp. or exp Blood Pressure/

200 (systolic$ adj2 pressure$).mp. [mp=title, abstract, original title, name of substance word, subject heading word, keyword heading word, protocol supplementary concept word, rare disease supplementary concept word, unique identifier]

201 (diastolic$ adj2 pressure$).mp. [mp=title, abstract, original title, name of substance word, subject heading word, keyword heading word, protocol supplementary concept word, rare disease supplementary concept word, unique identifier]

202 (pulse adj2 pressure).mp. [mp=title, abstract, original title, name of substance word, subject heading word, keyword heading word, protocol supplementary concept word, rare disease supplementary concept word, unique identifier]

203 exp hypertension/

204 ("blood pressure$" adj2 "high").mp. [mp=title, abstract, original title, name of substance word, subject heading word, keyword heading word, protocol supplementary concept word, rare disease supplementary concept word, unique identifier]

205 or/199-204

206 133 or 121

207 exp Cardiovascular Diseases/

208 87 or 207

209 67 and (123 or 133)

210 69 and (208 or 102 or 98 or 159 or 174 or 197 or 205)

211 209 or 210
